# Comparison of energy and nutrient intakes between weekdays and weekends in Japanese preschool children based on meal categories

**DOI:** 10.1265/ehpm.25-00247

**Published:** 2025-09-13

**Authors:** Atsuki Sakai, Katsushi Yoshita, Takako Takahashi, Tetsuko Okabe, Ruriko Sasaki, Hiromi Ishida, Hiromitsu Ogata, Mitsuhiko Hara, Yukiko Yoshioka, Miho Nozue, Tatsuaki Sakamoto, Sanae Ito, Nobuko Murayama

**Affiliations:** 1 Graduate School of Human Life and Ecology, Osaka Metropolitan University, Department of Nutrition, 3-3-138 Sugimoto, Sumiyoshi-ku, Osaka-shi, Osaka 558-8585, Japan; 2 School of Nursing and Nutrition, Tenshi College, Department of Nutrition, 3-1-30 Kita13-jo Higashi, Higashi-ku, Sapporo-shi, Hokkaido 065-0013, Japan; 3 Faculty of Human Life Science, Miyagi Gakuin Women’s University, Department of Food and Nutritional Science, 9-1-1 Sakuragaoka, Aoba-ku, Sendai-shi, Miyagi 981-8557, Japan; 4 Faculty of Nutrition, Kagawa Nutrition University, Department of Applied Nutrition, 3-9-21 Chiyoda, Sakado-shi, Saitama 350-0288, Japan; 5 Faculty of Human Ecology, Wayo Women’s University, Department of Health and Nutrition, 2-3-1 Konodai, Ichikawa-shi, Chiba 272-8533, Japan; 6 Faculty of Nutritional Science, Sagami Women’s University, Department of Nutritional Management, 2-1-1 Bunkyo, Minami-ku, Sagamihara-shi, Kanagawa 252-0383, Japan; 7 Faculty of Health Promotional Sciences, Tokoha University, Department of Health and Nutritional Sciences, 1230 Miyakodacho, Hamana-ku, Hamamatsu-shi, Shizuoka 431-2102, Japan; 8 Faculty of Environmental and Symbiotic Sciences, Prefectural University of Kumamoto, Department of Food & Health Sciences, 3-1-100 Tsukide, Higashi-ku, Kumamoto-shi, Kumamoto 862-8502, Japan; 9 Faculty of Medicine, University of the Ryukyus, Department of Health Sciences, 1076 Kiyuna, Ginowan-shi, Okinawa 901-2720, Japan; 10 Faculty of Human Life Studies, University of Niigata Prefecture, Department of Health and Nutrition, 471 Ebigase, Higashi-ku, Niigata-shi, Niigata 950-8680, Japan

**Keywords:** Preschool children, Energy and nutrient intake, Nursery school, Lunch

## Abstract

**Background:**

Meals are provided at nursery schools for Japanese preschool children, and this may result in different energy and nutrient intakes on weekdays and weekends. The purpose of this study was to obtain basic information for public nutrition policies in early childhood by examining differences in energy and nutrient intakes of preschool children between weekdays and weekends using meal categories such as breakfast, lunch, dinner, and snacks.

**Methods:**

Energy and nutrient intakes were examined in 761 Japanese preschool children (423 boys, 338 girls) aged 3–6 years attending childcare facilities in seven regions in Japan. Data collection was based on non-consecutive four-day dietary records (two weekdays, two weekend days) in 2019 or 2020. Energy and nutrient intake by meal category were compared using a generalized linear mixed model adjusted for demographic factors.

**Results:**

Total energy intake was significantly higher on weekdays for boys (1,478 vs. 1,415 kcal) and girls (1,349 vs. 1,296 kcal) (both P < 0.001). Weekday lunches had higher protein content and essential micronutrients such as potassium, iron, vitamins, and lower fat, saturated fatty acids, and salt, compared to weekend lunches. Similarly, weekday snacks also had significantly higher nutritional consumption for most nutrients compared to weekend equivalents.

**Conclusion:**

These findings suggest that meals at nursery schools, particularly lunch and snacks, result in more desirable nutrient intake in preschool children. On weekdays, children consume meals with higher nutrient density, possibly due to the role of dietitians in menu planning. However, some children still fail to meet the Dietary Reference Intakes for Japanese, indicating a need for improvements in nursery school and home meals. More communication is needed between nursery schools and families, including sharing menus and recipes is essential. The results of this study are of value for development of public health nutrition strategies targeting early childhood.

**Supplementary information:**

The online version contains supplementary material available at https://doi.org/10.1265/ehpm.25-00247.

## 1. Introduction

Eating habits formed during early childhood strongly influence dietary patterns in the school-age period and adulthood. Therefore, establishment of desirable eating habits in early childhood is critically important [1–3]. Acquiring an understanding of regular mealtimes and nutritionally balanced diets during early childhood is believed to contribute to future health maintenance and prevention of lifestyle-related diseases. The Fourth Basic Plan for Promotion of Shokuiku explicitly states that “The remarkable growth and development of children, babies and infants occurs during an important period of at a foundation of building good health for life” [4, 5].

According to the Children and Families Agency, 61.4% of preschool children aged 3 years and older attend childcare facilities such as nursery schools, centers for early childhood education and care (combined type and kindergarten type, ECEC centers) [6]. This increasing number of community-based childcare services and combined type ECEC centers from 2015 to 2024 reflect the growing demand for childcare outside the home [6]. Thus, nutritional intake of children is influenced by meals at home and at childcare facilities. Provision of meals and nutritional management at nursery schools aims to promote healthy growth and development, maintain and improve health and nutritional status, and enhance the quality of life of children [4, 7]. Nursery schools also position food education as an integral part of childcare, with the aim of cultivating the foundation for a healthy lifestyle, and this requires collaborative efforts among various professionals within the facility [8].

In a previous study, we found that the proportion of children whose habitual intake of vitamins and minerals fell below the Estimated Average Requirement (EAR) was lower on weekdays at childcare facilities compared to weekends at home [9]. Murakami et al. have also reported that the intake of most nutrients for Japanese preschool children aged 3–5 years fell below the Dietary Reference Intakes for Japanese (DRIs) [10]. However, the extent to which meals at nursery schools and other meals contribute to nutrient intake has not been clarified. Therefore, this study aimed to examine energy and nutrient intake by meal category on weekdays and weekends for children aged 3–6 years to obtain detailed information on dietary intake during early childhood.

## 2. Methods

This study was conducted according to the guidelines in the Declaration of Helsinki. All procedures involving study participants were approved by the ethics committees of the University of Niigata Prefecture, Japan (number 1918) and Osaka City University, Japan (number 19-32). Written informed consent was obtained from parents of all participants.

### 2.1. Participants

The participants were children in classes for 3- to 6-year-olds at 40 nursery schools (21 private and 19 public) located in one city in one of seven regions across Japan. These facilities consisted of 31 licensed nursery schools and 9 certified children centers, both of which are government-approved childcare institutions, and all provided lunch as part their daily program. In this study, all these facilities are collectively referred to as ‘nursery schools’. Of the 2,703 children invited to participate, consent was obtained for 850 (participation rate: 31.4%). A total of 761 participants were included in the final analysis (effective response rate: 28.1%), after exclusion of 49 children who did not complete the four-day food survey, 3 without physical measurement data, and 17 with incomplete questionnaire responses. In total, there were 423 boys and 338 girls, and the numbers of children in each of the seven cities were: City A 42 boys, 33 girls; City B 53 boys, 44 girls; City C 93 boys, 57 girls; City D 63 boys, 44 girls; City E 80 boys, 66 girls; City F 47 boys, 46 girls; and City G 45 boys, 48 girls.

### 2.2. Physical measurements

Height and weight measurements taken within one month prior to the dietary survey were collected from each facility. The obesity index was calculated from using the following formula:

$$\begin{array}{rl} & \text{Obesity index (\%)}\\ & \;=(\text{Weight (kg)}-\text{Standard weight for height (kg)})\\ & \;/\text{Standard weight for height (kg)}\times 100 \end{array}$$

Participants were classified as: underweight tendency: <−15%, “normal”: −15% to <15%, and “overweight tendency”: ≥15% [11].
Standard weight for height was calculated using age- and sex-specific equations derived from national growth surveys [12, 13].
For ages 3–5 years:
Boys: 0.00206 × height^2^ − 0.1166 × height + 6.5237
Girls: 0.00249 × height^2^ − 0.1858 × height + 9.0360
For age 6 years:
Boys: 0.461 × height − 32.382
Girls: 0.458 × height − 32.079

### 2.3. Dietary surveys

A dietary survey was conducted over four days, including two non-consecutive weekdays and two weekend days from October to December in 2019 or 2020, using weighed record and estimated record methods. These methods have been used in several previous studies involving young children and have also served as a reference method for validating other dietary assessment tools [10, 14, 15]. In this study, “weekdays” were defined as days on which children attended nursery school and ate lunch there, while “weekend days” were defined as all other days.

### 2.4. Meals provided at nursery schools

Meals provided at nursery schools included lunches and snacks planned by registered dietitians. To assess intake on survey days, an estimated record method was used. Childcare workers recorded the proportion of food consumed by each child for each dish category of the lunch: staple food, main dish, side dish, and soup. To minimize variation between surveyors, instruction manuals were distributed in advance and explanations were provided to the person in charge. The same recording forms were used across all facilities to standardize the procedure. Intake was calculated based on these records and the actual lunch and snack menus provided. For example, if the planned serving amount was set at 100% and a child’s intake was recorded as 90%, the planned amount was multiplied by 0.9. Conversely, if a child had seconds and intake reached 150%, the amount was multiplied by 1.5.

### 2.5. Meals provided at home (outside nursery schools)

For meals provided at home, parents were asked to weigh and record foods using the weighing record method. They were provided with an instruction sheet illustrating the food recording procedure, one food record form per day (four forms in total), and measuring equipment including a digital scale (Tanita, Tokyo; Digital Scale KJ-114 or equivalent), a measuring cup (Kagawa Nutrition University, Tokyo; 200 ml measuring cup), and measuring spoons/spatulas (Kagawa Nutrition University; 15 ml, 5 ml, and 1 ml). The food record forms were designed differently for weekdays and weekend days. Weekday forms included sections for breakfast, dinner, and snacks (excluding lunch at nursery school), while weekend forms included sections for breakfast, lunch, dinner, and snacks. Snacks were further categorized into six times: before breakfast, before lunch before dinner, after breakfast, after lunch, and after dinner. The total snack intake was calculated as the sum of snacks consumed across all time point. If meals were eaten out or ready-made foods were consumed and it was difficult to measure individual food items or dishes, parents were asked to estimate portion sizes, attach food packaging, or provide details such as the place of purchase, menu items, and estimated consumption as thoroughly as possible. All completed food record forms, including those from nursery schools and home settings, were reviewed by registered dietitians, who followed up on missing or unclear information and made corrections as necessary.

### 2.6. Eating habits survey

A self-administered questionnaire on eating habits was distributed with the dietary survey. This questionnaire included items on children’s food allergies, parental educational background, annual household income, household size, and other factors.

### 2.7. Calculating energy and nutrient intake

Energy and nutrient intake were calculated using the nutrition calculation software “*Shokuji Shirabe*” developed by the National Institutes of Biomedical Innovation, Health and Nutrition. Daily intake values were estimated based on the Standard Tables of Food Composition in Japan, 2015 (Seventh Revised Version) [16]. “*Shokuji Shirabe*” accounts for changes in nutrient content and food weight during cooking using cooking codes, allowing for more accurate estimation of energy and nutrient intake from the foods and beverages consumed. We assessed energy and 16 nutrients, with reference to the priority guidelines in the DRIs (2010 Edition) and “Meal Planning Using the Dietary Reference Intakes in Child Welfare Facilities” [17, 18]. Nutrient intake was calculated for total daily intake and separately for breakfast, lunch, dinner, and snacks. For participants who skipped a meal, the intake for that meal was treated as zero. Total and meal category nutrient intakes were adjusted per 1,000 kcal.

### 2.8. Analysis methods

Associations between differences in energy intake between weekdays and weekends and variables such as age, height, weight, obesity index, EI, and socioeconomic status were examined. Participants were categorized into three groups based on the difference between their mean weekday and weekend energy intake: >100 kcal decrease, ±100 kcal (no change), and >100 kcal increase. Continuous variables were analyzed using the Kruskal-Wallis test and categorical variables were analyzes using the χ^2^ test. Statistical analyses were conducted separately by sex to compare energy and nutrient intakes between weekdays and weekends. This is because significant differences were observed in total energy intake and energy intake by meal categories between boys and girls on both weekdays and weekends. In addition, previous studies have reported sex-related differences in dietary or life behaviors during early childhood [19]. The equivalent income (EI) was calculated by dividing household income by the square root of the number of household members. To compare energy and macronutrient distribution, nutrient intake per 1,000 kcal intake between weekdays and weekends, a generalized linear mixed model was used. Individual identification numbers were used to designate subjects in the data structure. The model was adjusted for age, obesity index, presence of allergies in children, number of siblings, EI, and parental educational attainment as fixed effects, while region was included as a random effect. Results are presented as estimated adjusted means with 95% confidence intervals (CI). All statistical analyses were performed using SPSS Statistics 30.0 (IBM Japan, Tokyo). The significance level was set at 5%.

## 3. Results

### 3.1. Participants

Table 1 shows the characteristics of the participants. The median values (25th, 75th percentiles) for age, height, weight, obesity index, and EI were 4.0 years, 105.1 cm, 17.2 kg, −0.4%, and 21,775 US dollars, respectively. No significant associations were found between changes in energy intake between weekdays and weekends and age, height, weight, obesity index, or EI. While a significant difference in region was observed among three groups, no significant differences were found in sex distribution, evaluation of obesity index, presence of food allergies, or parental educational background.

**Table 1:** Participant Characteristics by change in energy intake between weekdays and weekends

		**Total (n = 761)**		**>100 kcal decrease (n = 209)**		**±100 kcal (n = 204)**		**>100 kcal increase (n = 348)**		

		**Median**	**25th, 75th** **Percentiles**	**Median**	**25th, 75th** **Percentiles**	**Median**	**25th, 75th** **Percentiles**	**Median**	**25th, 75th** **Percentiles**	**P value**
Age	(years)	4.0	(4.0, 5.0)	5.0	(4.0, 5.0)	4.0	(4.0, 5.0)	4.0	(4.0, 5.0)	0.399
Height	(cm)	105.1	(100.4, 111.0)	106.3	(100.0, 112.2)	105.1	(100.4, 110.1)	104.9	(100.5, 110.7)	0.235
Weight	(kg)	17.2	(15.4, 19.1)	17.3	(15.5, 19.8)	17.1	(15.5, 18.8)	17.0	(15.3, 19.0)	0.341
Obesity index*	(%)	−0.4	(−5.2, 5.0)	−0.3	(−5.4, 5.5)	−0.6	(−4.0, 4.3)	−0.4	(−5.5, 5.4)	0.866
Equivalent income**	(US $)	21,775	(16,482, 25,145)	12,084	(17,407, 25,145)	19,832	(15,075, 25,138)	21,775	(16,482, 26,800)	0.102

		**n**	**(%)**	**n**	**(%)**	**n**	**(%)**	**n**	**(%)**	**P value**

Sex										
Boys		423	(55.6)	116	(55.5)	111	(54.4)	196	(56.3)	0.909
Girls		338	(44.4)	93	(44.5)	93	(45.6)	152	(43.7)	
Region										
A		75	(9.9)	16	(7.7)	32	(15.7)	27	(7.8)	0.004
B		97	(12.7)	20	(9.6)	22	(10.8)	55	(15.8)	
C		150	(19.7)	40	(19.1)	34	(16.7)	76	(21.8)	
D		107	(14.1)	43	(20.6)	29	(14.2)	35	(10.1)	
E		146	(19.2)	36	(17.2)	40	(19.6)	70	(20.1)	
F		93	(12.2)	27	(12.9)	20	(9.8)	46	(13.2)	
G		93	(12.2)	27	(12.9)	27	(13.2)	39	(11.2)	
Evaluation of obesity index										
underweight tendency		12	(1.6)	3	(1.4)	2	(1.0)	7	(2.0)	0.351
normal		715	(94.0)	193	(92.3)	197	(96.6)	325	(93.4)	
overweight tendency		34	(4.5)	13	(6.3)	5	(2.4)	16	(4.6)	
Food allergies										
Yes		58	(7.6)	19	(9.1)	10	(4.9)	29	(8.3)	0.219
No		703	(92.4)	190	(90.9)	194	(95.1)	319	(91.7)	
Educational background of father										
Less than high school		35	(4.6)	12	(5.7)	11	(5.4)	12	(3.5)	0.802
High school		206	(27.1)	56	(26.8)	57	(27.9)	93	(26.7)	
Vocational school		131	(17.2)	39	(18.7)	35	(17.2)	57	(16.4)	
Junior college		18	(2.4)	7	(3.3)	4	(2.0)	7	(2.0)	
University/graduate school		371	(48.8)	95	(45.5)	97	(47.5)	179	(51.4)	
Educational background of mother										
Less than high school		11	(1.4)	3	(1.4)	5	(2.5)	3	(0.8)	0.586
High school		143	(18.8)	45	(21.5)	31	(15.2)	67	(19.3)	
Vocational school		170	(22.3)	49	(23.5)	46	(22.5)	75	(21.5)	
Junior college		128	(16.8)	31	(14.8)	40	(19.6)	57	(16.4)	
University/graduate school		309	(40.6)	81	(38.8)	82	(40.2)	146	(42.0)	

Participants were classified into three groups according to the difference between mean energy intake on weekdays and weekends: >100 kcal decrease, ±100 kcal, and >100 kcal increase.

P values were calculated using the Kruskal-Wallis test for continuous variables and χ^2^ test for categorical variables.

*Obesity index (%) = [body weight (kg) − standard body weight for height (kg)]/[standard body weight for height (kg)].

**Equivalent income was calculated by dividing household income by the square root of the number of household members. For reference, an exchange rate 1 Japanese Yen = 0.0067 US Dollars was used.

### 3.2. Comparison of energy and nutrient intake between weekdays and weekends

The estimated adjusted mean energy intake was 1,478 kcal on weekdays and 1,415 kcal on weekends for boys, and 1,349 kcal on weekdays and 1,296 kcal on weekends for girls, with significantly higher intake on weekdays for both boys and girls (both P < 0.001). Regarding macronutrient energy distribution, energy from protein was significantly higher on weekdays for both sexes (P < 0.001). On weekdays, energy from fat was significantly lower for boys (P < 0.001) and energy from carbohydrates was significantly lower for girls (P < 0.001). Intake of protein, potassium, calcium, magnesium, iron, zinc, copper, vitamin A, vitamin B_1_, vitamin B_2_, and dietary fiber was significantly higher on weekdays for both sexes (P < 0.001). Carbohydrate intake was significantly lower on weekdays for girls (P < 0.001). Salt equivalent intake was significantly lower on weekdays for both sexes (P < 0.001) (Table 2).

**Table 2:** Comparison of energy and nutrient intake between weekdays and weekends

		**Boys (n = 423)**					**Girls (n= 338)**				
	
		**Weekdays**		**Weekends**		**P value**	**Weekdays**		**Weekends**		**P value**
Energy	(kcal)	1,478	(1,423–1,534)	1,416	(1,361–1,471)	<0.001	1,350	(1,286–1,413)	1,298	(1,234–1,361)	<0.001
Energy from protein	(%)	14.6	(14.2–15.0)	13.5	(13.1–13.9)	<0.001	14.7	(14.3–15.2)	13.3	(12.8–13.8)	<0.001
Energy from fat	(%)	28.6	(27.4–29.7)	29.6	(28.5–30.7)	<0.001	28.9	(27.6–30.2)	28.9	(27.6–30.3)	0.861
Energy from carbohydrate	(%)	56.8	(55.6–58.1)	56.9	(55.6–58.2)	0.913	56.4	(54.9–57.9)	57.8	(56.3–59.3)	<0.001
Energy from SFA*	(%)	9.6	(9.1–10.1)	9.9	(9.4–10.4)	0.018	9.6	(8.9–10.2)	9.7	(9.0–10.3)	0.482
Protein	(g/1,000 kcal)	36.4	(35.4–37.5)	33.8	(32.8–34.8)	<0.001	36.9	(35.7–38.0)	33.2	(32.0–34.4)	<0.001
Fat	(g/1,000 kcal)	31.8	(30.5–33.0)	32.9	(31.6–34.2)	<0.001	32.1	(30.6–33.6)	32.2	(30.7–33.6)	0.862
Carbohydrates	(g/1,000 kcal)	138.8	(135.5–142.1)	139.1	(135.8–142.4)	0.715	138.2	(134.4–142.0)	141.7	(137.9–145.5)	<0.001
Potassium	(mg/1,000 kcal)	1,234	(1,184–1,284)	1,052	(1,001–1,102)	<0.001	1,316	(1,259–1,374)	1,100	(1,043–1,158)	<0.001
Calcium	(mg/1,000 kcal)	365	(338–393)	292	(265–319)	<0.001	382	(350–414)	297	(264–329)	<0.001
Magnesium	(mg/1,000 kcal)	125	(120–130)	104	(99–109)	<0.001	130	(125–136)	106	(101–112)	<0.001
Iron	(mg/1,000 kcal)	3.9	(3.7–4.1)	3.4	(3.2–3.6)	<0.001	3.7	(3.5–3.9)	3.2	(3.0–3.4)	<0.001
Zinc	(mg/1,000 kcal)	4.4	(4.2–4.5)	3.9	(3.8–4.0)	<0.001	4.4	(4.2–4.5)	3.8	(3.7–4.0)	<0.001
Copper	(mg/1,000 kcal)	0.54	(0.52–0.56)	0.48	(0.46–0.50)	<0.001	0.54	(0.52–0.56)	0.49	(0.47–0.51)	<0.001
Vitamin A	(µgRAE/1,000 kcal)	323	(284–362)	237	(198–276)	<0.001	342	(303–381)	270	(231–310)	<0.001
Vitamin B_1_	(mg/1,000 kcal)	0.54	(0.51–0.57)	0.49	(0.46–0.52)	<0.001	0.55	(0.52–0.56)	0.49	(0.47–0.51)	<0.001
Vitamin B_2_	(mg/1,000 kcal)	0.64	(0.60–0.68)	0.57	(0.53–0.61)	<0.001	0.68	(0.63–0.73)	0.60	(0.56–0.65)	<0.001
Vitamin C	(mg/1,000 kcal)	38	(32–43)	38	(33–44)	0.551	45	(39–51)	46	(40–52)	0.325
SFA	(g/1,000 kcal)	10.65	(10.08–11.23)	10.98	(10.41–11.56)	0.018	10.64	(9.94–11.34)	10.75	(10.05–11.45)	0.484
Dietary fiber	(g/1,000 kcal)	9.3	(8.8–9.7)	8.4	(8.0–8.8)	<0.001	9.5	(9.0–10.0)	8.7	(8.2–9.1)	<0.001
Salt equivalent	(g/1,000 kcal)	4.1	(3.9–4.3)	4.6	(4.4–4.8)	<0.001	3.9	(3.7–4.2)	4.5	(4.2–4.8)	<0.001

*SFA: Saturated fatty acid

Estimated adjusted mean (95% confidence interval), nutrient intakes are energy-adjusted per 1,000 kcal.

Generalized linear mixed model adjusted for age, obesity index, presence of children’s allergies, number of siblings, equivalent income, and paternal educational attainment as fixed effects, and region as a random effect.

### 3.3. Breakfast

The number and percentage of breakfast skipping occasions over the two weekdays were 6 boys (0.7%) and 1 girl (0.1%), and over the two weekend days were 18 boys (2.1%) and 8 girls (1.2%).

For breakfast energy intake, boys consumed 336 kcal on weekdays and 338 kcal on weekends, with no significant difference. In contrast, girls consumed 283 kcal on weekdays and 293 kcal on weekends, with significantly higher intake on weekends. Regarding other nutrients, intake of carbohydrate, magnesium copper, and dietary fiber was significantly higher on weekdays for both sexes. For girls, energy from fat and intake of fat was significantly higher on weekends (Table 3).

**Table 3:** Comparison of energy and nutrient intake between weekdays and weekends (breakfast)

		**Boys (n = 423)**					**Girls (n = 338)**				
	
		**Weekdays**		**Weekends**		**P value**	**Weekdays**		**Weekends**		**P value**
Energy	(kcal)	336	(311–360)	337	(313–362)	0.740	282	(255–309)	292	(265–320)	0.040
Energy from protein	(%)	12.6	(11.8–13.4)	12.5	(11.7–13.3)	0.568	12.8	(11.9–13.7)	12.8	(11.9–13.7)	0.946
Energy from fat	(%)	25.2	(22.7–27.6)	25.9	(23.4–28.4)	0.214	25.1	(22.1–28.2)	26.4	(23.4–29.5)	0.040
Energy from carbohydrates	(%)	61.4	(58.3–64.4)	59.3	(56.3–62.4)	0.002	61.2	(57.7–64.6)	58.8	(55.3–62.3)	0.002
Energy from SFA*	(%)	9.6	(8.5–10.8)	9.9	(8.7–11.0)	0.323	9.0	(7.6–10.5)	9.4	(8.0–10.9)	0.132
Protein	(g/1,000 kcal)	31.6	(29.6–33.5)	31.3	(29.4–33.3)	0.568	32.0	(29.7–34.3)	32.0	(29.7–34.3)	0.946
Fat	(g/1,000 kcal)	28.0	(25.2–30.7)	28.7	(26.0–31.5)	0.214	27.9	(24.5–31.3)	29.4	(26.0–32.8)	0.04
Carbohydrates	(g/1,000 kcal)	151.8	(144.0–159.6)	147.1	(139.3–154.9)	0.005	151.8	(142.9–160.7)	146.0	(137.1–154.9)	0.003
Potassium	(mg/1,000 kcal)	1,011	(908–1114)	1,008	(905–1110)	0.867	1,094	(975–1,213)	1,103	(984–1,221)	0.727
Calcium	(mg/1,000 kcal)	365	(302–428)	355	(292–418)	0.400	373	(302–445)	369	(297–440)	0.73
Magnesium	(mg/1,000 kcal)	109	(100–118)	105	(96–114)	0.029	113	(101–125)	107	(95–118)	0.013
Iron	(mg/1,000 kcal)	3.5	(3.0–4.0)	3.5	(3.0–4.0)	0.968	3.4	(2.8–4.1)	3.2	(2.6–3.9)	0.129
Zinc	(mg/1,000 kcal)	3.6	(3.2–4.0)	3.6	(3.2–4.0)	0.823	3.8	(3.5–4.1)	3.7	(3.4–4.0)	0.042
Copper	(mg/1,000 kcal)	0.50	(0.46–0.54)	0.47	(0.43–0.51)	0.005	0.49	(0.43–0.55)	0.45	(0.39–0.50)	<0.001
Vitamin A	(µgRAE/1,000 kcal)	215	(161–269)	213	(159–267)	0.867	306	(228–383)	318	(240–396)	0.426
Vitamin B_1_	(mg/1,000 kcal)	0.45	(0.39–0.51)	0.45	(0.39–0.51)	0.842	0.45	(0.34–0.56)	0.48	(0.36–0.59)	0.088
Vitamin B_2_	(mg/1,000 kcal)	0.65	(0.56–0.75)	0.63	(0.53–0.72)	0.186	0.64	(0.54–0.75)	0.67	(0.57–0.78)	0.118
Vitamin C	(mg/1,000 kcal)	33	(22–44)	33	(22–44)	0.956	42	(30–55)	44	(32–57)	0.463
SFA	(g/1,000 kcal)	10.72	(9.43–12.00)	10.99	(9.71–12.27)	0.323	10.01	(8.40–11.61)	10.49	(8.89–12.10)	0.132
Dietary fiber	(g/1,000 kcal)	8.9	(8.2–9.7)	8.6	(7.8–9.4)	0.049	8.8	(7.7–9.8)	8.3	(7.3–9.3)	0.031
Salt equivalent	(g/1,000 kcal)	4.0	(3.6–4.4)	4.0	(3.6–4.4)	0.929	4.2	(3.6–4.7)	4.2	(3.7–4.7)	0.889

*SFA: Saturated fatty acid

Estimated adjusted mean (95% confidence interval), nutrient intakes are energy-adjusted per 1,000 kcal.

Generalized linear mixed model adjusted for age, obesity index, presence of children’s allergies, number of siblings, equivalent income, and paternal educational attainment as fixed effects, and region as a random effect.

### 3.4. Lunch

None of the children skipped lunch on either weekdays or weekends.

For lunch energy intake, boys consumed significantly more on weekends, while for girls there was no significant difference. Regarding macronutrient energy distribution, energy from protein was significantly higher and energy from fat was significantly lower on weekdays for boys and girls. Energy from carbohydrates was significantly higher on weekdays for boys. On weekdays, both sexes had significantly higher intake of protein, potassium, magnesium, iron, zinc, copper, vitamin A, vitamin B_1_, vitamin C and dietary fiber; and significantly lower intake of fat, saturated fatty acids (SFA), and salt equivalent (Table 4).

**Table 4:** Comparison of energy and nutrient intake between weekdays and weekends (lunch)

		**Boys (n = 423)**					**Girls (n = 338)**				
	
		**Weekdays**		**Weekends**		**P value**	**Weekdays**		**Weekends**		**P value**
Energy	(kcal)	409	(385–432)	424	(401–447)	0.024	389	(365–413)	385	(361–410)	0.557
Energy from protein	(%)	16.0	(15.4–16.5)	14.1	(13.5–14.6)	<0.001	16.5	(15.9–17.1)	14.1	(13.5–14.8)	<0.001
Energy from fat	(%)	24.3	(22.7–25.8)	27.5	(26.0–29.1)	<0.001	24.6	(22.7–26.5)	26.4	(24.6–28.3)	0.001
Energy from carbohydrates	(%)	59.8	(58.0–61.5)	58.4	(56.6–60.2)	0.014	58.9	(56.8–60.9)	59.4	(57.4–61.5)	0.334
Energy from SFA*	(%)	5.6	(5.0–6.2)	7.9	(7.3–8.5)	<0.001	6.1	(5.4–6.9)	8.0	(7.2–8.7)	<0.001
Protein	(g/1,000 kcal)	39.9	(38.5–41.3)	35.2	(33.8–36.5)	<0.001	41.3	(39.7–42.9)	35.3	(33.7–36.9)	<0.001
Fat	(g/1,000 kcal)	27.0	(25.2–28.7)	30.6	(28.6–32.3)	<0.001	27.4	(25.3–29.5)	29.4	(27.3–31.5)	0.001
Carbohydrates	(g/1,000 kcal)	145.4	(141.0–149.8)	141.5	(137.1–146.0)	0.005	143.4	(138.4–148.5)	144.1	(139.1–149.2)	0.635
Potassium	(mg/1,000 kcal)	1,360	(1,299–1,420)	956	(896–1,017)	<0.001	1,457	(1,383–1,530)	1,006	(933–1,080)	<0.001
Calcium	(mg/1,000 kcal)	231	(207–256)	226	(202–251)	0.516	236	(207–265)	215	(186–244)	0.007
Magnesium	(mg/1,000 kcal)	146	(140–153)	95	(88–101)	<0.001	153	(144–161)	100	(92–109)	<0.001
Iron	(mg/1,000 kcal)	4.5	(4.3–4.7)	3.1	(2.9–3.3)	<0.001	4.5	(4.3–4.8)	3.2	(3.0–3.5)	<0.001
Zinc	(mg/1,000 kcal)	4.8	(4.6–5.1)	3.8	(3.6–4.0)	<0.001	5.0	(4.7–5.2)	3.8	(3.6–4.1)	<0.001
Copper	(mg/1,000 kcal)	0.66	(0.63–0.68)	0.47	(0.45–0.50)	<0.001	0.67	(0.64–0.69)	0.50	(0.47–0.52)	<0.001
Vitamin A	(µgRAE/1,000 kcal)	483	(404–562)	245	(166–324)	<0.001	435	(364–507)	215	(144–287)	<0.001
Vitamin B_1_	(mg/1,000 kcal)	0.59	(0.54–0.64)	0.49	(0.44–0.54)	<0.001	0.66	(0.60–0.73)	0.53	(0.47–0.60)	<0.001
Vitamin B_2_	(mg/1,000 kcal)	0.49	(0.44–0.54)	0.54	(0.49–0.59)	0.003	0.55	(0.49–0.61)	0.57	(0.51–0.62)	0.467
Vitamin C	(mg/1,000 kcal)	49	(43–55)	39	(33–45)	<0.001	51	(43–59)	46	(39–54)	0.049
SFA	(g/1,000 kcal)	6.25	(5.59–6.92)	8.74	(8.07–9.40)	<0.001	6.80	(5.97–7.63)	8.86	(8.03–9.69)	<0.001
Dietary fiber	(g/1,000 kcal)	12.2	(11.6–12.9)	9.1	(8.5–9.8)	<0.001	12.6	(11.9–13.4)	9.7	(8.9–10.5)	<0.001
Salt equivalent	(g/1,000 kcal)	3.7	(3.3–4.1)	5.5	(5.2–5.9)	<0.001	3.7	(3.1–4.2)	6.0	(5.5–6.6)	<0.001

*SFA: Saturated fatty acid

Estimated adjusted mean (95% confidence interval), nutrient intakes are energy-adjusted per 1,000 kcal.

Generalized linear mixed model adjusted for age, obesity index, presence of children’s allergies, number of siblings, equivalent income, and paternal educational attainment as fixed effects, and region as a random effect.

We also compared the proportion of total daily intake accounted for by lunch between weekdays and weekends. The proportion of fat and salt equivalent intake at lunch was lower on weekdays, whereas the proportion of most vitamins and minerals was higher on weekdays (Additional file 1).

### 3.5. Dinner

The number and percentage of dinner skipping occasions over the two weekdays were 1 boy (0.1%) and 1 girl (0.1%), and over the two weekend days were 10 boys (1.2%) and 4 girls (0.6%).

There were no significant differences in energy intake at dinner between weekdays and weekends for either boys or girls. For boys, intake of copper and dietary fiber was significantly higher on weekdays. For girls, energy from protein, fat, and SFA, as well as intake of protein, fat, potassium, calcium, magnesium, iron, vitamin B_1_, vitamin B_2_, vitamin C, SFA, and dietary fiber were significantly higher on weekdays (Table 5).

**Table 5:** Comparison of energy and nutrient intake between weekdays and weekends (dinner)

		**Boys (n = 423)**					**Girls (n = 338)**				
	
		**Weekdays**		**Weekends**		**P value**	**Weekdays**		**Weekends**		**P value**
Energy	(kcal)	449	(419–479)	437	(407–467)	0.094	400	(368–432)	390	(358–423)	0.200
Energy from protein	(%)	16.3	(15.5–17.2)	16.1	(15.2–16.9)	0.366	16.4	(15.5–17.4)	15.9	(15.0–16.9)	0.040
Energy from fat	(%)	30.1	(28.2–32.0)	29.7	(27.8–31.6)	0.47	30.9	(28.7–33.1)	29.1	(26.9–31.3)	0.004
Energy from carbohydrates	(%)	53.4	(51.2–55.6)	52.9	(50.7–55.2)	0.451	53.0	(50.4–55.5)	54.8	(52.3–57.4)	0.006
Energy from SFA*	(%)	8.5	(7.8–9.2)	8.6	(7.9–9.3)	0.756	8.3	(7.4–9.1)	7.8	(6.9–8.6)	0.024
Protein	(g/1,000 kcal)	40.8	(38.6–42.9)	40.2	(38.1–42.3)	0.366	41.1	(38.7–43.5)	39.8	(37.4–42.2)	0.04
Fat	(g/1,000 kcal)	33.4	(31.3–35.5)	33.0	(30.9–35.1)	0.47	34.3	(31.9–36.8)	32.4	(29.9–34.8)	0.004
Carbohydrates	(g/1,000 kcal)	127.7	(122.0–133.4)	126.8	(121.1–132.5)	0.565	127.5	(121.1–133.9)	131.8	(125.4–138.2)	0.011
Potassium	(mg/1,000 kcal)	1,185	(1,101–1,269)	1,145	(1,061–1,229)	0.068	1,224	(1,130–1,318)	1,152	(1,058–1,246)	<0.001
Calcium	(mg/1,000 kcal)	236	(203–269)	236	(203–269)	0.993	264	(229–299)	238	(203–273)	0.002
Magnesium	(mg/1,000 kcal)	123	(113–132)	118	(109–128)	0.121	130	(119–140)	121	(111–131)	<0.001
Iron	(mg/1,000 kcal)	3.8	(3.0–4.5)	3.9	(3.1–4.6)	0.695	4.0	(3.7–4.3)	3.8	(3.5–4.1)	0.013
Zinc	(mg/1,000 kcal)	4.9	(4.7–5.2)	4.8	(4.6–5.1)	0.274	4.8	(4.5–5.1)	4.6	(4.3–4.9)	0.108
Copper	(mg/1,000 kcal)	0.58	(0.55–0.51)	0.55	(0.52–0.58)	0.005	0.59	(0.54–0.63)	0.58	(0.54–0.63)	0.843
Vitamin A	(µgRAE/1,000 kcal)	311	(226–396)	306	(221–391)	0.858	288	(216–361)	286	(213–358)	0.907
Vitamin B_1_	(mg/1,000 kcal)	0.61	(0.55–0.66)	0.60	(0.54–0.65)	0.595	0.54	(0.48–0.61)	0.50	(0.44–0.56)	0.009
Vitamin B_2_	(mg/1,000 kcal)	0.56	(0.51–0.61)	0.54	(0.49–0.59)	0.095	0.56	(0.51–0.62)	0.53	(0.47–0.58)	0.006
Vitamin C	(mg/1,000 kcal)	45	(35–54)	42	(33–52)	0.376	48	(39–56)	43	(35–52)	0.063
SFA	(g/1,000 kcal)	9.48	(8.69–10.26)	9.55	(8.76–10.34)	0.755	9.17	(8.27–10.08)	8.62	(7.71–9.52)	0.024
Dietary fiber	(g/1,000 kcal)	9.9	(9.1–10.63)	9.4	(8.7–10.1)	0.033	10.4	(9.6–11.3)	10.0	(9.1–10.8)	0.034
Salt equivalent	(g/1,000 kcal)	5.9	(5.4–6.5)	6.0	(5.4–6.5)	0.829	5.4	(4.8–6.0)	5.5	(4.9–6.1)	0.456

*SFA: Saturated fatty acid

Estimated adjusted mean (95% confidence interval), nutrient intakes are energy-adjusted per 1,000 kcal.

Generalized linear mixed model adjusted for age, obesity index, presence of children’s allergies, number of siblings, equivalent income, and paternal educational attainment as fixed effects, and region as a random effect.

### 3.6. Snacks

The number and percentage of children who did not consume snacks on either of the two weekdays were 5 boys (0.6%) and 1 girl (0.1%), while on the two weekend days, 103 boys (12.2%) and 57 girls (8.4%).

Energy intake from snacks was significantly higher on weekdays than on weekends for both boys and girls. Regarding macronutrient energy distribution, energy from protein and fat was significantly higher on weekdays for both sexes. Similarly, intake of protein, fat, potassium, calcium, magnesium, iron, vitamin A, vitamin B_1_, vitamin B_2_, SFA, dietary fiber, and salt equivalent was significantly higher on weekdays for both sexes. While intake of carbohydrates and vitamin C was significantly higher on weekends (Table 6).

**Table 6:** Comparison of energy and nutrient intake between weekdays and weekends (snacks)

		**Boys (n = 423)**					**Girls (n = 338)**				
	
		**Weekdays**		**Weekends**		**P value**	**Weekdays**		**Weekends**		**P value**
Energy	(kcal)	285	(255–314)	217	(188–246)	<0.001	279	(246–312)	230	(197–263)	<0.001
Energy from protein	(%)	12.4	(11.7–13.1)	6.5	(5.8–7.2)	<0.001	12.0	(11.2–12.8)	6.1	(5.3–6.9)	<0.001
Energy from fat	(%)	29.4	(27.0–31.8)	22.1	(19.7–24.5)	<0.001	30.9	(27.9–33.9)	24.5	(21.5–27.4)	<0.001
Energy from carbohydrates	(%)	54.5	(51.0–58.0)	56.1	(52.6–59.6)	0.114	52.9	(49.0–56.9)	57.0	(53.1–60.9)	<0.001
Energy from SFA*	(%)	14.0	(12.7–15.3)	9.7	(8.4–11.0)	<0.001	14.8	(13.2–16.5)	11.1	(9.5–12.8)	<0.001
Protein	(g/1,000 kcal)	30.9	(29.1–32.7)	16.2	(14.4–18.0)	<0.001	30.0	(28.0–31.9)	15.3	(13.3–17.2)	<0.001
Fat	(g/1,000 kcal)	32.6	(30.0–35.3)	24.5	(21.9–27.2)	<0.001	34.3	(31.0–37.6)	27.2	(23.9–30.5)	<0.001
Carbohydrates	(g/1,000 kcal)	136.4	(127.1–145.7)	143.5	(134.2–152.8)	0.007	132.3	(122.0–142.6)	145.5	(135.2–155.8)	<0.001
Potassium	(mg/1,000 kcal)	1,601	(1,446–1,757)	1,199	(1,044–1,354)	<0.001	1,480	(1,330–1,631)	1,027	(876–1,178)	<0.001
Calcium	(mg/1,000 kcal)	781	(715–847)	395	(330–461)	<0.001	740	(667–813)	345	(272–418)	<0.001
Magnesium	(mg/1,000 kcal)	127	(117–138)	90	(79–100)	<0.001	119	(109–130)	82	(72–92)	<0.001
Iron	(mg/1,000 kcal)	4.2	(3.4–4.9)	3.1	(2.3–3.8)	<0.001	2.6	(2.0–3.3)	1.7	(1.1–2.4)	<0.001
Zinc	(mg/1,000 kcal)	4.1	(2.9–5.3)	3.7	(2.5–4.9)	0.234	3.1	(2.1–4.2)	2.4	(1.3–3.4)	0.023
Copper	(mg/1,000 kcal)	0.43	(0.38–0.48)	0.40	(0.35–0.45)	0.055	0.38	(0.33–0.43)	0.35	(0.30–0.40)	0.035
Vitamin A	(µgRAE/1,000 kcal)	255	(146–363)	178	(70–286)	<0.001	341	(254–429)	264	(176–351)	0.003
Vitamin B_1_	(mg/1,000 kcal)	0.46	(0.40–0.53)	0.32	(0.26–0.39)	<0.001	0.45	(0.40–0.49)	0.31	(0.27–0.36)	<0.001
Vitamin B_2_	(mg/1,000 kcal)	1.13	(0.98–1.28)	0.64	(0.49–0.80)	<0.001	1.00	(0.90–1.09)	0.50	(0.41–0.59)	<0.001
Vitamin C	(mg/1,000 kcal)	53	(22–83)	81	(50–112)	0.005	37	(15–59)	56	(34–78)	0.003
SFA	(g/1,000 kcal)	15.51	(14.06–16.96)	10.76	(9.31–12.20)	<0.001	16.46	(14.63–18.28)	12.37	(10.54–14.19)	<0.001
Dietary fiber	(g/1,000 kcal)	5.4	(4.6–6.2)	4.5	(3.7–5.3)	<0.001	5.4	(4.5–6.3)	4.8	(3.9–5.7)	0.019
Salt equivalent	(g/1,000 kcal)	2.2	(2.0–2.4)	1.3	(1.1–1.5)	<0.001	2.1	(1.9–2.3)	1.1	(0.9–1.3)	<0.001

*SFA: Saturated fatty acid

Estimated adjusted mean (95% confidence interval), nutrient intakes are energy-adjusted per 1,000 kcal.

Generalized linear mixed model adjusted for age, obesity index, presence of children’s allergies, number of siblings, equivalent income, and paternal educational attainment as fixed effects, and region as a random effect.

## 4. Discussion

This study compared nutrient intake by meal category between weekdays (when children ate lunch at nursery school) and weekends in preschool children attending nursery school in multiple regions of Japan. Most children had obesity indices within the normal range, and their energy intake was broadly consistent with national data [20]. However, a small proportion showed underweight or overweight tendencies, indicating the need for individualized monitoring.

Nutrient intake during lunch was significantly better on weekdays than weekends, with higher protein and micronutrient levels and lower fat and salt, highlighting the value of nursery school lunches. These findings suggest that lunches provided at nursery schools play an essential role in promoting desirable nutritional intake in preschool children.

Weekday snacks contributed to higher nutrient intake overall. While fat intake from snacks slightly exceeded DRI limits [21], the nutrients composition was generally more appropriate than that on weekends. Intake of most nutrients, except for vitamin C, was higher on weekdays, suggesting that differences in snack quality between weekdays and weekends may influence overall nutrient intake. In addition, the proportions of recommended nutrients such as vitamins, minerals, and dietary fiber consumed at lunch were higher on weekdays than on weekends, with most approaching 30% of total daily intake. When combined with snacks, the intake of certain nutrients accounted for approximately 50% of total daily intake. On weekdays, lunches and snacks consumed by children at nursery schools are nutritionally managed, as the menus are planned by registered dietitians. Thus, children were consuming meals with high nutrient density on weekdays. This may have contributed to the lower proportion of children not meeting the DRIs on weekdays, as reported in previous studies [9].

Furthermore, in this study, the timing of snack consumption was assessed in detail. The most common timing was after lunch, accounting for approximately 70% of total snack occasions on weekdays and about 55% on weekends. In the present study, snacks were treated as a single category; however, future studies should examine how differences in the timing and quantity of snack consumption between weekdays and weekends may influence total dietary intake and the observed weekdays-weekends differences. Additionally, differences in energy intake patterns between weekdays and weekends were also observed across regions. Although no clear trend was identified to explain these variations, this finding highlights the potential influence of regional or local factors on children’s dietary behaviors. Future studies are warranted to explore how such regional characteristics may contribute to differences in eating patterns.

The higher rate of breakfast and dinner skipping on weekends may partly explain the lower nutrient intake on those days. Further research is needed to examine the specific impact of meal skipping. Previous research has identified that some preschool children who do not meet the DRIs on weekdays, indicating the need to improve the quality of meals both at nursery schools and at home [9]. On weekdays, intake of potassium, calcium, iron, vitamin B_1_, vitamin B_2_, and dietary fiber was significantly higher at dinner. To support more desirable nutrient intake patterns, particularly for breakfast on weekdays and meals at weekends, we believe it is important for nursery schools to provide nutritional information and practical guidance to families. Because preschool children have limited stomach capacity, appropriate snacks are essential for meeting nutritional needs. Studies in the US support the role of nutrient-dense snacks in improving diet quality [22]. Therefore, snacks between the three main meals play an important role for preschool children, who often struggle to consume adequate amounts of nutrients. Providing families with information to help incorporate appropriate snacks into children’s diets at home is also essential. Previous studies on information delivery have suggested that printed materials have higher viewing rates than online formats. Therefore, distributing flyers that include information on the content, menus, and recipes of lunches and snacks provided at nursery schools may be an effective strategy [23]. Strengthening communication between nursery schools and families through nutrition education tailored to each household is also a key priority. The effectiveness of nutrition education in nursery schools has been reported to improve when families are actively involved [24]. Conversely, especially during early childhood, appropriate nutrition education may not be achieved unless there are changes in the family’s awareness of nutrition and overall lifestyle. Therefore, when implementing nutrition education programs in nursery schools, it is essential to provide support that encourages family participation, along with tailored assistance that takes into account diverse family circumstances, such as single-parent households or parents’ employment status. Additionally, it has been reported that dietary patterns in early childhood differ by sex, with girls more likely than boys to choose healthier foods [19]. Moreover, the presence of older siblings has been shown to influence young children’s dietary behaviors and overall lifestyle [25, 26]. Therefore, when implementing nutrition education for preschool children, it is important to tailor programs to individual characteristics, taking into consideration sex differences and family environment.

According to the Report on Public Health Administration and Services in 2023, about 66% of childcare facilities have dietitians, while only about 25% employ registered dietitians [27]. Nozue et al. found that private nursery schools with dietitians on staff or enhanced nutrition management certifications were more likely to continuously assess and improve both meal provision and nutrition education for parents [28, 29]. Given the critical role of nursery schools in fostering healthy eating habits and providing nutritional support to families, proper implementation of nutritional management is essential. Therefore, increasing the employment of dietitians in nursery schools is considered key strategy for improving nutritional management and supporting healthy dietary patterns in preschool children.

The strength of this study lies in its design, which involved selecting target areas by dividing Japan into multiple regional blocks and conducted dietary surveys over four non-consecutive days including two weekdays and two weekend days. This approach reduced the likelihood of extreme regional bias in this sample. Additionally, including both weekdays and two weekends in the survey period helped mitigate bias related to day-to-day variations in dietary intake. However, the study still has several limitations. First, the participation rate was relatively low at 28.8%. It is possible that the families who participated in the study had greater health awareness or more time available, which may limit the generalizability of the findings. Seconds, the participants were restricted to children at nursery schools, and caution is warranted when applying these results to children attending kindergarten or receiving care at home. In addition, the participating nursery schools varied in their types and educational philosophies, and the impact of each facility’s approach to nutrition education was not evaluated in this study. It is also important to consider that the educational policies and philosophies of nursery schools may influence children’s dietary behaviors. Third, dietary intake was assessed using the food record method. Although the food record methods are considered a gold standard among dietary assessment methods, it imposes a substantial burden on participants and may lead to reporting bias, such as intentional or unintentional misreporting of food intake. In addition, underreporting of intake has been shown in previous studies [18, 30]. Notably, weekday lunches were recorded by nursery school staffs, whereas all other meals including all meals on weekends were recorded by parents. Given this discrepancy in recorders, caution is warranted when interpreting differences in energy and nutrient intake between weekdays and weekends, as the accuracy and consistency of recording may vary. The survey was conducted in 2020 in five regions during the COVID-19 pandemic, and changes in children’s dietary habits may have occurred at this time. For instance, Sakamoto et al. found that the frequency of sweet food and beverage consumption increased among preschool children during the state of emergency in Japan. Therefore, potential pandemic-related influences, particularly on weekend snack consumption, should be considered when interpreting the findings [31].

Finally, because multiple statistical analyses were conducted across various nutrients and meal categories, the possibility of type I error due to multiple comparisons cannot be excluded.

## 5. Conclusion

This study compared nutrient intake by meal category between weekdays and weekends in preschool children aged 3 to 6 years attending nursery school. The results showed that nutrient intake at lunch was generally higher on weekdays, and both energy and most nutrient intakes from snacks were significantly higher on weekdays as well. These findings suggest that lunches and snacks provided at nursery schools play a substantial role in promoting desirable nutrient intake patterns in preschool children. To further improve overall dietary intake, particularly for meals at home on weekends, close collaboration among dietitians, nursery schools, and families is essential. Efforts should focus on information sharing and nutrition education initiatives tailored to support healthy eating habits beyond the nursery school setting.

## Abbreviations

ECEC: Early Childhood Education and Care
PDCA: Plan-Do-Check-Action
EAR: Estimated Average Requirement
DRIs: Dietary Reference Intakes for Japanese
SD: Standard Deviation
CI: Confidence Interval
DG: Dietary Goal for preventing lifestyle-related disease

## Supplementary information

**Additional file 1:** **Fig. 1.** Distribution of energy and major nutrient intake by meal categories on Weekdays and Weekends (boys). **Fig. 2.** Distribution of energy and major nutrient intake by meal categories on Weekdays and Weekends (girls).

**Additional file 2:** **Supplementary Table 1.** Comparison of energy and nutrient intake between weekdays and weekends. **Supplementary Table 2.** Comparison of energy and nutrient intake between weekdays and weekends (breakfast). **Supplementary Table 3.** Comparison of energy and nutrient intake between weekdays and weekends (lunch). **Supplementary Table 4.** Comparison of energy and nutrient intake between weekdays and weekends (dinner). **Supplementary Table 5.** Comparison of energy and nutrient intake between weekdays and weekends (snacks).

## Declarations

### Availability of data and materials

Data cannot be shared for privacy or ethical reasons.
