# Supplementary material for: Comparison of energy and nutrient intakes between weekdays and weekends in Japanese preschool children based on meal categories
Source: Environ Health Prev Med. 2025 Sep 13;30:71. doi: 10.1265/ehpm.25-00247 (PMC12436065; doi:10.1265/ehpm.25-00247)
Supplement: Supplementary file 1 — Additional file 1: Fig. 1. Distribution of energy and major nutrient intake by meal categories on Weekdays and Weekends (boys). Fig. 2. Distribution of energy and major nutrient intake by meal categories on Weekdays and Weekends (girls). [file ehpm-30-071-s001.pdf]

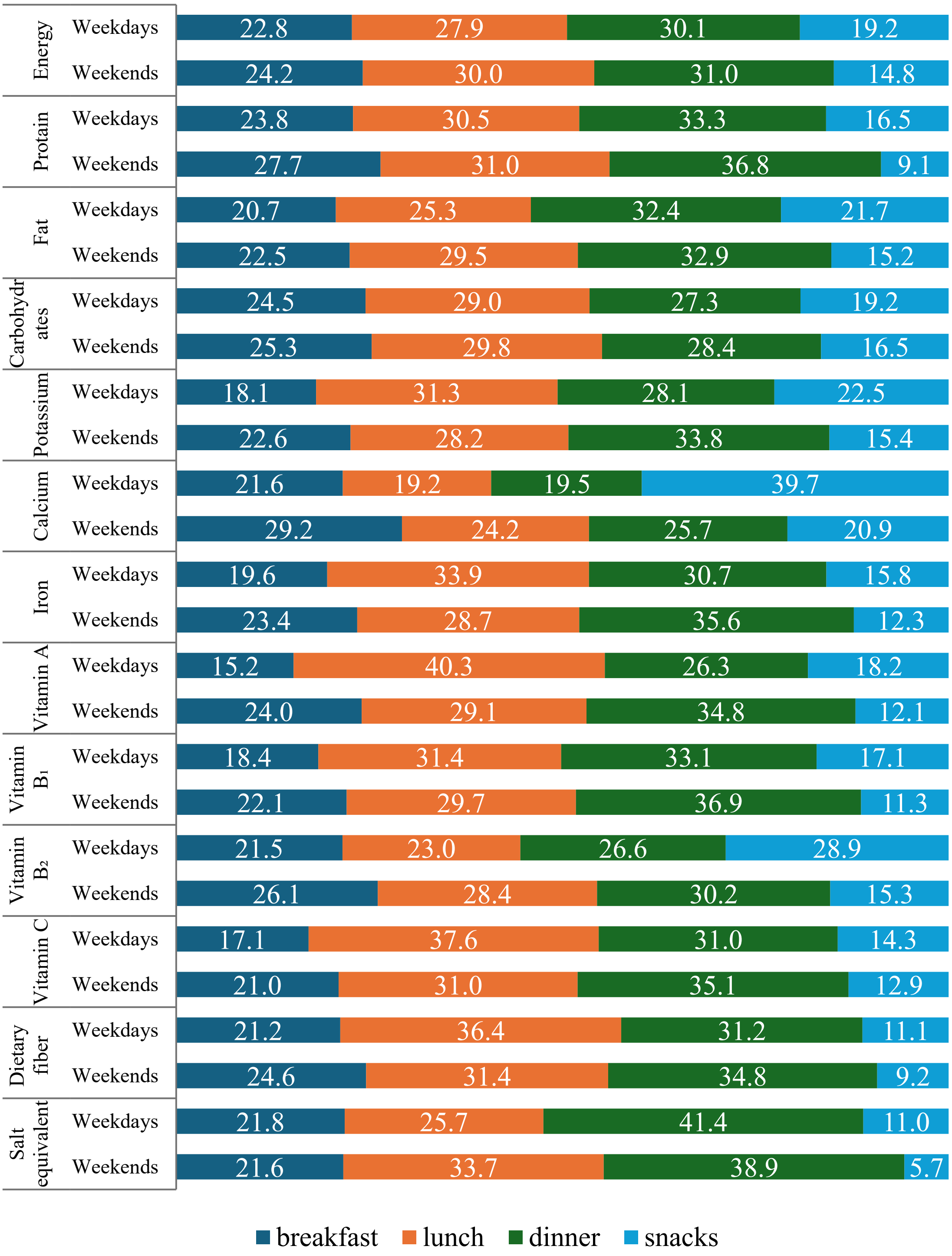

Fig 1. Distribution of energy and major nutrient intake by meal categories on Weekdays and Weekends (boys)

n=423

Estimated adjusted means (%) were calculated using generalized linear mixed model, adjusting for age, obesity index, presence of children's allergies, equivalent income, and parental educational attainments fixed effects, and region as a random effect.

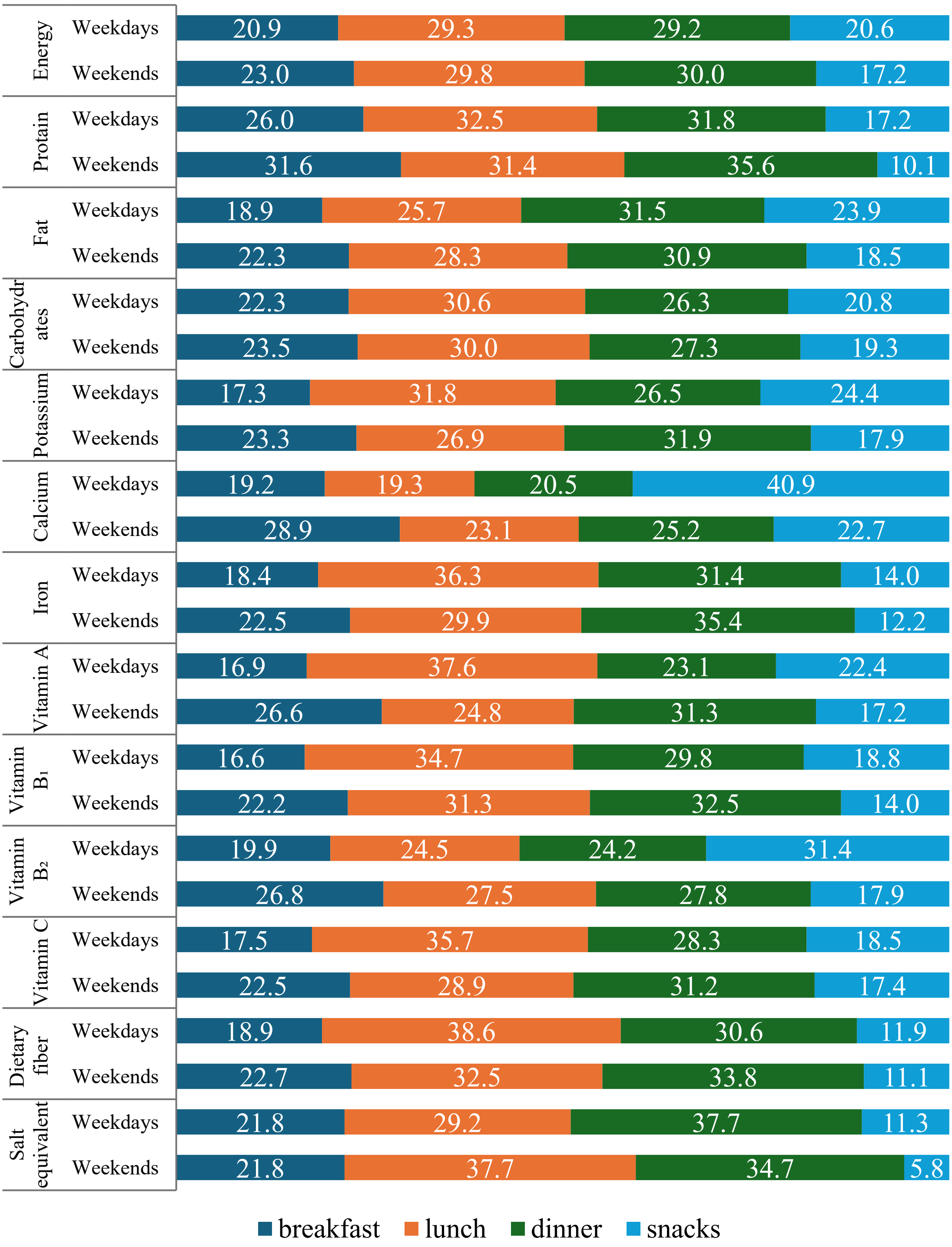

Fig 2. Distribution of energy and major nutrient intake by meal categories on Weekdays and Weekends (girls)

n=338

Estimated adjusted means (%) were calculated using generalized linear mixed model, adjusting for age, obesity index, presence of children's allergies, equivalent income, and parental educational attainments fixed effects, and region as a random effect.
